# Comparison of Distal Radius Fracture Outcomes in Older Adults Stratified by Chronologic vs Physiologic Age Managed With Casting vs Surgery

**DOI:** 10.1001/jamanetworkopen.2022.55786

**Published:** 2023-02-13

**Authors:** Mayank Jayaram, Hao Wu, Alfred P. Yoon, Robert L. Kane, Lu Wang, Kevin C. Chung

**Affiliations:** 1 Department of Surgery, University of Michigan Medical School, Ann Arbor; 2 University of Michigan School of Public Health, Ann Arbor; 3 Section of Plastic Surgery, Department of Surgery, University of Michigan Hospital, Ann Arbor; 4 Section of Plastic Surgery, Department of Surgery, The Medical University of South Carolina, Charleston; 5 University of Michigan School of Public Health, Ann Arbor; 6 Department of Surgery, Section of Plastic Surgery, University of Michigan Medical School, Ann Arbor

## Abstract

**Importance:**

Casting is recommended for adults older than 65 years with distal radius fractures (DRFs) because similar long-term outcomes are achieved regardless of treatment. However, physiologically younger adults could benefit from operative DRF management despite advanced chronologic age.

**Objective:**

To examine how chronologic age compares with measures of physiologic age in DRF treatment recovery.

**Design, Setting, and Participants:**

This retrospective secondary analysis of the Wrist and Radius Injury Surgical Trial (WRIST) was performed from May 1 to August 31, 2022. WRIST was a 24-center randomized clinical trial that enrolled participants older than 60 years with unstable DRFs from April 1, 2012, to December 31, 2016.

**Interventions:**

Participants selected casting or surgery. Patients who selected surgery were randomly assigned to volar lock plating, percutaneous pinning, or external fixation. Participants were stratified by chronologic age, number of comorbidities, and activity status.

**Main Outcomes and Measures:**

The primary outcome was Michigan Hand Outcomes Questionnaire (MHQ) score assessed at 6 weeks, 3 months, 6 months, and 1 year. Partial correlation (PC) analysis adjusted for confounding.

**Results:**

The final cohort consisted of 293 participants (mean [SD] age, 71.1 [8.89] years; 255 [87%] female; 247 [85%] White), with 109 receiving casting and 184 receiving surgery. Increased chronologic age was associated with increased MHQ scores in the surgery group at all time points but decreased MHQ scores in the casting group at 12 months (mean [SD] score, −0.46 [0.21]; *P* = .03). High activity was associated with improved MHQ scores in the surgical cohort at 6 weeks (mean [SD] score, 12.21 [5.18]; PC = 0.27; *P* = .02) and 12 months (mean [SD] score, 13.25 [5.77]; PC = 0.17; *P* = .02). Comorbidities were associated with decreased MHQ scores at all time points in the casting group. Clinically significant differences in MHQ scores were associated with low physical activity, 4 or more comorbidities, or increased age by 15 years.

**Conclusions and Relevance:**

In this retrospective secondary analysis of WRIST, chronologic age was not associated with functional demand. These findings suggest that physicians should counsel active older adults with few comorbidities on earlier return to daily activities after surgery compared with casting.

**Trial Registration:**

ClinicalTrials.gov Identifier: NCT01589692

## Key Points

**Question:**

Is recovery after distal radius fracture managed with casting vs surgery different in patients stratified by physiologic vs chronologic age?

**Findings:**

This secondary analysis of the Wrist and Radius Injury Surgical Trial demonstrated that older adults with high preinjury activity levels and few comorbidities had clinically significant improvement in short-term patient-reported outcomes after surgical treatment of distal radius fractures.

**Meaning:**

These findings suggest that surgeons should place emphasis on a patient’s baseline activity level and number of comorbidities to guide operative treatment decisions of distal radius fractures in older adults, even if these individuals have an advanced chronologic age.

## Teaser

This secondary analysis of the Wrist and Radius Injury Surgical Trial examines differences in outcomes after distal radius fracture managed with casting vs surgery in patients stratified by physiologic vs chronologic age.

## Introduction

Distal radius fractures (DRFs) account for 18% of fractures in the active elderly population.^1^ The estimated cost of treating DRFs in older adults in 2025 is $600 million.^2^ Distal radius fractures are treated nonoperatively with casting or surgically with volar locking plates (VLPs), percutaneous pinning (PP), or external fixation (EF). Although numerous studies^3,4^ have compared operative with nonoperative treatment of DRFs, no consensus has been reached regarding optimal management in the geriatric population. Evidence from randomized clinical trials suggests long-term patient-reported outcomes are comparable, regardless of treatment received or postreduction radiographic alignment.^5,6^ However, key differences among DRF treatments are evident in their short-term outcomes. Compared with other treatments, VLPs are associated with significantly better patient-reported outcomes in the early recovery period and enable faster return to mobility.^6^

In 2020, the American Academy of Orthopedic Surgeons published appropriate use criteria for DRF management.^7,8,9^ These guidelines provide evidence that operative management of DRFs in patients older than 65 years does not improve long-term (>1 year) patient-reported outcomes and suggest that casting should be the preferred treatment for DRFs in older adults. However, these guidelines use chronologic age as a proxy for estimating functional demand and do not consider that older adults in the US are living longer and leading active lifestyles well into older age. A commentary in *JAMA Surgery*^10^ advocated the use of physiologic age to make important decisions on the treatment of older adults with DRFs. The principle of physiologic age operates under the assumption that an individual’s health deteriorates at varying rates. Therefore, by aggregating factors related to health status, such as comorbidities and functional demand, we can estimate the biologic function or physiologic age of an individual. According to the Centers for Disease Control and Prevention, average life expectancy in the US has increased 2 years since 1999.^11^ In addition, older adults have a longer disability-free life expectancy and are maintaining activities of daily living for longer periods.^12^ These factors challenge traditional beliefs that casting elderly patients with DRFs is appropriate with the assumption that the elderly population has lower functional demands.

Elderly patients with the same chronologic age can vary greatly in activity level and frailty.^13,14,15^ For example, sedentary patients with multiple comorbidities may prefer casting to avoid surgical risks, even with the need for longer immobilization. However, highly active and independent patients may derive greater benefit from the faster recovery afforded by VLPs. Identifying preoperative factors with the greatest association with positive outcomes can reconcile conflicting conclusions in the DRF literature and can assist health care professionals in using lifestyle and health-related factors to personalize treatment decisions for older patients with DRFs. Prior analyses^16,17,18,19^ of patients with DRFs have demonstrated that increased activity status and reduced comorbidities may be associated with patient-reported recovery and reduction in complication rates after DRF treatment. However, these studies^16,17,18,19^ are frequently designed using a retrospective database that cannot directly compare physiologic age with chronologic age. The purpose of our study was to investigate whether physiologic or chronologic age has a stronger association with patient-reported outcomes after DRF treatment.

## Methods

### Study Population

This study was a secondary analysis of prospectively collected data from the Wrist and Radius Injury Surgical Trial (WRIST). WRIST was a multicenter randomized clinical trial that recruited adults older than 60 with unstable DRFs from 24 health systems across the US, Canada, and Singapore.^6^ Participants opted for nonoperative management (casting) or were randomly assigned to 1 of 3 surgical cohorts (VLPs, PP, or EF). Individuals with open fractures, bilateral DRFs, concurrent upper extremity fractures or ligament injuries, previous DRF of the same wrist, or comorbidities that prevented surgical treatment were excluded from this cohort (Figure). All patient characteristics were self-reported. Because most distal radius fractures occur in white women, data on race and ethnicity were collected to mirror the general population and allow for generalizability. Patient-reported outcomes were assessed using the Michigan Hand Outcomes Questionnaire (MHQ), a 37-item legacy questionnaire in hand surgery validated for many musculoskeletal conditions.^20,21,22,23^ The MHQ is scored from 0 to 100, with higher scores representing better outcomes. Participants completed the MHQ at 6 weeks, 3 months, 6 months, and 1 year after treatment. Previous studies^24,25,26,27^ have demonstrated that the minimal clinically important difference (MCID) for the MHQ is between 8 and 13 points. Therefore, an MCID threshold of 8 was selected to indicate clinically significant changes in MHQ score over time. All participants completed written informed consent forms before enrollment. Institutional review board approval was received at all sites, and this study followed the Strengthening the Reporting of Observational Studies in Epidemiology (STROBE) reporting guidelines for cohort studies. The WRIST trial protocol is available in Supplement 1.

**Figure.**
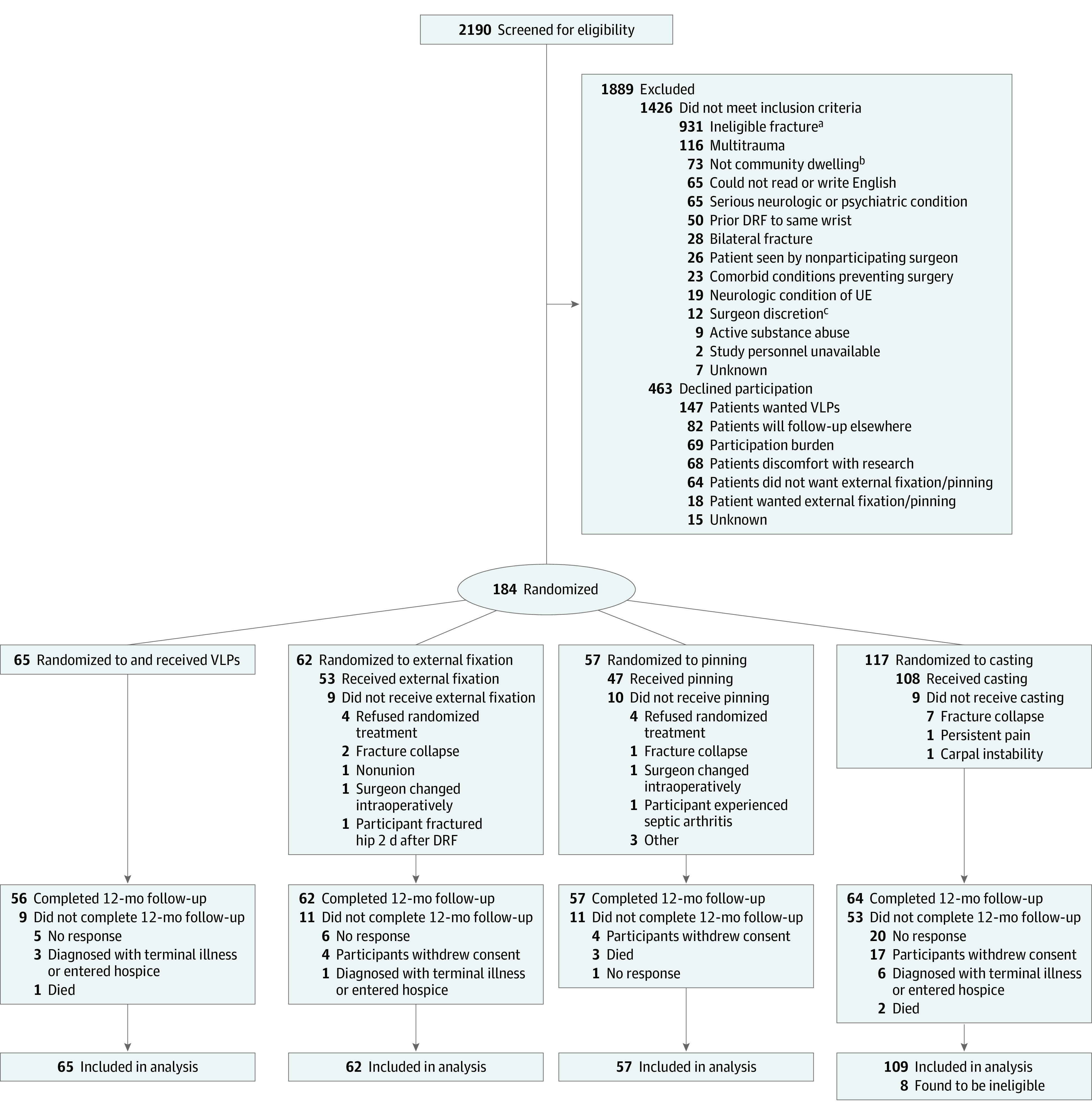
Eligibility Criteria and Randomization Process for the Wrist and Radius Injury Surgical Trial (WRIST) Cohort ^a^Ineligible fractures include nondisplaced fractures, open fractures, and those that are not amenable to treatment with all 3 surgical methods. ^b^Noncommunity dwelling patients include those who reside in a nursing home or other institutional setting. ^c^These were cases in which the patient was too anxious, upset, or angry for the surgeon to feel comfortable broaching the subject of a clinical trial or in which the surgeon felt that discussing the trial was inappropriate for some other reason. DRF indicates distal radius fracture; UE, upper extremity; VLPs, volar locking plates.

### Cohort Stratification

For this analysis, participants were stratified into 2 cohorts: casting and surgery (including VLPs, PP, and EF). We hypothesized that activity level and number of comorbidities would have the greatest association with DRF outcomes. Therefore, all participants were stratified by number of comorbidities and activity status. The Rapid Assessment of Physical Activity (RAPA) measured functional status as a proxy of physiologic age. RAPA is a validated 9-item questionnaire to assess physical activity level in adults older than 50 years.^18,28^ Patients are placed into 3 categories: sedentary, underactive, and regularly active. For this analysis, RAPA scores of 3 were categorized as high activity, whereas RAPA scores of 1 or 2 were categorized as low activity. To determine preinjury number of comorbidities, all participants completed the Self-Administered Comorbidity Questionnaire (SCQ) at baseline. The SCQ is a validated 12-item scale developed for patients with limited medical knowledge.^29^

### Statistical Analysis

All patient characteristics are summarized as means (SDs) for continuous variables or numbers (percentages) for categorical variables. Group differences between surgery and casting were compared using a 2-sample *t* test for continuous variables or a Pearson χ^2^ test or Fisher exact test for categorical variables.^30,31^ Linear mixed-effects models evaluated associations between MHQ summary score and each independent variable (chronologic age, activity level, and number of comorbidities), controlling for potential confounders, such as smoking, sex, and race. We used random effects to account for within-subject correlations among the repeated measurements.^32^ Partial correlations (PCs) were calculated to determine the variation in MHQ summary score that could be attributed to each independent variable, removing the effect of other variables in the model.^33^ All PCs are measured on a scale of −1 to +1, with values closer to 0 indicating poor correlation. A log-linked generalized linear model investigated the association between total complications at 1 year with number of comorbidities, activity level, and chronologic age. The linear mixed-effects model was used for a subgroup analysis comparing VLPs with casting. The number of comorbidities was a count variable, chronologic age was a continuous variable, and activity level was a categorical variable divided into low and high activity. An a priori significance level was set at a 1-sided *P* ≤ .05. All analyses were performed using R, version 4.2.0 and R Studio, version 2022.06.0 (R Foundation for Statistical Computing).

## Results

### Participant Characteristics

The final cohort consisted of 293 participants (mean [SD] age, 71.1 [8.89] years; 255 [87%] female; 247 [85%] White), with 109 receiving casting and 184 receiving surgery (Table 1). Age and race were significantly different between the surgical and casting groups. The mean (SD) age was 68.4 (7.2) years in the surgical group vs 75.6 (9.6) years in the casting group (*P* < .001). The surgical cohort consisted of 7 (4%) Asian, 11 (6%) Black, and 163 (89%) White participants compared with the casting group, which consisted of 16 (15%) Asian, 6 (6%) Black, and 84 (77%) White participants (*P* = .004). A greater proportion of participants in the surgical group had high activity levels compared with the casting group (82 [45%] vs 30 [28%], *P* = .004) (Table 1). There were no statistically significant differences in number of comorbidities, smoking, sex, education, employment status, income, or dominant hand injury.

**Table 1.** Patient Characteristics in the Final Study Cohort^a^

Characteristic	Overall (N = 293)	Casting cohort (n = 109)	Surgical cohort (n = 184)	*P* value
Age, mean (SD), y	71.1 (8.9)	75.6 (9.6)	68.4 (7.2)	<.001
Activity level				
Low	181 (62)	79 (72)	102 (55)	.004
High	112 (38)	30 (28)	82 (45)	
No. of comorbidities, mean (SD)	3.53 (2.44)	3.78 (2.67)	3.38 (2.29)	.18
Smoking				
Never	155 (53)	57 (52)	98 (53)	.45
Current	28 (10)	9 (8)	19 (10)	
Former				
≤10 y	11 (4)	2 (2)	9 (5)	
>10 y	99 (34)	41 (38)	58 (32)	
Sex				
Male	38 (13)	17 (16)	21 (11)	.30
Female	255 (87)	92 (84)	163 (89)	
Race				
American Indian/Alaska Native	1 (1)	0 (0)	1 (1)	.004
Asian	23 (8)	16 (15)	7 (4)	
Black	17 (6)	6 (6)	11 (6)	
Pacific Islander/Hawaii Native	1 (1)	1 (1)	0	
White	247 (85)	84 (77)	163 (89)	
Missing	1 (NA)	0 (NA)	1 (NA)	
≥2 Races or other^b^	3 (1)	2 (2)	1 (1)	
Dominant hand injured	119 (42)	45 (44)	74 (42)	.76
Missing	13 (NA)	6 (NA)	7 (NA)	
Educational level				
Less than high school graduate	38 (13)	19 (18)	19 (11)	.35
High school diploma or GED	70 (24)	27 (25)	43 (24)	
Vocational or technical school	16 (6)	3 (3)	13 (7)	
Some college or associate’s degree	71 (25)	27 (25)	44 (24)	
College graduate	41 (14)	13 (12)	28 (16)	
Professional or graduate degree	52 (18)	19 (18)	33 (18)	
Missing	5 (NA)	1 (NA)	4 (NA)	
Employment status				
Full time	46 (17)	11 (11)	35 (20)	.39
Part time	34 (12)	12 (12)	22 (12)	
Retired	180 (65)	71 (71)	109 (62)	
Disability	7 (3)	2 (2)	5 (3)	
Unemployed	10 (4)	4 (4)	6 (3)	
Missing	16 (NA)	9 (NA)	7 (NA)	
Income, $				
<10 000	19 (7)	10 (10)	9 (5)	.06
10 000-19 999	43 (16)	21 (22)	22 (13)	
20 000-29 999	38 (14)	15 (15)	23 (14)	
30 000-39 999	35 (13)	16 (16)	19 (11)	
40 000-49 999	30 (11)	10 (10)	20 (12)	
50 000-59 999	20 (8)	4 (4)	16 (10)	
60 000-69 999	22 (8)	8 (8)	14 (8)	
>70 000	57 (22)	13 (13)	44 (26)	
Missing	29 (NA)	12 (NA)	17 (NA)	

Abbreviations: GED, General Educational Development; NA, not applicable.

^a^ Data are presented as number (percentage) of study participants unless otherwise indicated. *P* ≤ .05 was considered statistically significant.

^b^ The other category includes East Indian, Persian, and Indian (non-American) descent.

### Surgical Cohort

In the surgical cohort, activity level had a statistically and clinically significant effect on MHQ scores in the early recovery period (6 weeks). At 6 weeks, highly active individuals scored 12 points higher on the MHQ compared with less active individuals (Table 2). After adjusting for other variables in the model, activity level also had the highest partial correlation with MHQ score at 6 weeks (PC = 0.27, *P* ≤ .001) and 3 months (PC = 0.23, *P* = .007) of follow-up when compared with chronologic age (PC = 0.05 at 6 weeks, *P* = .58; PC = 0.20 at 3 months, *P* = .02) and number of comorbidities (PC = −0.12 at 6 weeks, *P* = .16; PC = −0.12 at 3 months, *P* = .18) (Table 3). After 6 months, activity level had no statistical or clinically significant association with overall MHQ score after adjusting for PC. Number of comorbidities had no significant association with MHQ score at any time point. However, the negative association between MHQ score and number of comorbidities was significant at 6 months (PC = −0.23, *P* = .009) and 12 months (PC = −0.18, *P* = .04) after treatment. Greater chronologic age was associated with a statistically significant increase in MHQ score at 3 months (by 0.62 points, *P* = .02) and 6 months (by 0.68 points, *P* = .006), with a significant PC of 0.20 (*P* = .02) at 3 months and 0.19 (*P* = .03) at 6 months (Table 3). Assuming an MCID of 8, clinically significant changes in MHQ score occur in individuals who are 15 years older than baseline. For example, a 75-year-old individual is expected to score approximately 8 points higher on the MHQ compared with a 60-year-old individual.

**Table 2.** Association of Chronologic Age, Number of Comorbidities, and Activity Level With MHQ Score at Each Time Point^a^

Time point	Chronologic age		No. of comorbidities		Activity level	
	MHQ Score, Mean (SE)	*P* value	MHQ Score, Mean (SE)	*P* value	MHQ Score, Mean (SE)	*P* value
**Surgery**						
6 wk	0.58 (0.25)	.02	0.66 (1.04)	.52	12.21 (5.18)	.02
3 mo	0.62 (0.25)	.01	1.08 (1.06)	.31	9.03 (5.23)	.08
6 mo	0.68 (0.25)	.006	0.46 (1.10)	.67	6.27 (5.45)	.25
12 mo	0.60 (0.25)	.02	0.49 (1.18)	.68	13.25 (5.77)	.02
**Casting**						
6 wk	−0.25 (0.19)	.20	−1.73 (0.82)	.04	0.05 (4.31)	.99
3 mo	−0.15 (0.20)	.44	−1.99 (0.83)	.02	0.24 (4.35)	.95
6 mo	−0.30 (0.20)	.14	−2.43 (0.87)	.005	−2.64 (4.60)	.56
12 mo	−0.46 (0.21)	.03	−1.86 (0.94)	.05	−6.35 (4.94)	.19

Abbreviation: MHQ, Michigan Hand Outcomes Questionnaire.

^a^ The model is controlled for smoking, sex, race, and treatment type. *P* ≤ .05 was considered statistically significant.

**Table 3.** Partial Correlation Between MHQ Scores and Chronologic Age, Number of Comorbidities, and Activity Level at Each Time Point, Adjusted for Other Covariates in the Model^a^

Time point	Chronologic age		No. of comorbidities		Activity level	
	Partial correlation	*P* value	Partial correlation	*P* value	Partial correlation	*P* value
**Surgery**						
6 wk	0.05	.58	−0.12	.16	0.27	.001
3 mo	0.20	.02	−0.12	.18	0.23	.007
6 mo	0.19	.03	−0.23	.009	0.10	.24
12 mo	0.07	.42	−0.18	.04	0.17	.06
**Casting**						
6 wk	−0.02	.89	−0.27	.02	0.02	.86
3 mo	−0.15	.20	−0.28	.02	−0.01	.92
6 mo	−0.35	.006	−0.32	.01	−0.08	.53
12 mo	−0.41	.007	−0.25	.11	−0.17	.27

Abbreviation: MHQ, Michigan Hand Outcomes Questionnaire.

^a^ *P* ≤ .05 was considered statistically significant.

### Casting Cohort

In the casting cohort, activity level had no clinically or statistically significant association with MHQ score at any time point (Table 2). Each additional comorbidity was associated with decreased MHQ scores by 1.73 points at 6 weeks (*P* = .01), 1.99 points at 3 months (*P* = .01), 2.43 points at 6 months (*P* = .005), and 1.86 points at 12 months (*P* = .04) (Table 2). Therefore, a clinically significant decrease in MHQ scores of 8 points would be associated with 4 or more additional comorbidities. The number of comorbidities also accounted for a large portion of MHQ score variation after casting with a PC of −0.27 (*P* = .02) at 6 weeks, −0.28 (*P* = .02) at 3 months and −0.32 (*P* = .01) at 6 months (Table 3). Greater chronologic age was associated with decreased MHQ scores at 12 months (−0.46 points, *P* = .03) (Table 2) after casting. As a result, clinically significant decreases in MHQ score are observed in individuals who are 15 years older than baseline. Statistically significant PCs between chronologic age and MHQ score were present at 6 months (−0.35, *P* = .006) and 12 months (−0.41, *P* = .007) (Table 3) after casting.

### Complications

Age, comorbidities, and activity status had no significant association with number of complications at 1-year follow-up (Table 4).These results were consistent for both the surgical and casting cohorts.

**Table 4.** Total Complication Rates at 1 Year After Stratifying by Chronologic Age, Number of Comorbidities, and Activity Level

Parameter	Relative risk (95% CI)	*P* value
Surgery		
Chronologic age	0.99 (0.97-1.01)	.31
No. of comorbidities	1.02 (0.96-1.07)	.54
Activity level	1.07 (0.84-1.38)	.58
Casting		
Chronologic age	1.02 (0.99-1.03)	.09
No. of comorbidities	1.05 (0.99-1.12)	.09
Activity level	1.25 (0.90-1.71)	.18

### Subgroup Analysis

In the VLP cohort, greater activity level was associated with a statistically and clinically significant improvement in MHQ scores at 3 months (12.46, *P* = .03) and 12 months (16.70, *P* = .006) compared with casting (eTable in Supplement 2). However, the number of comorbidities and chronologic age were not associated with MHQ scores in the VLP cohort.

## Discussion

To our knowledge, this is the first study that analyzed data from a prospective, multicenter randomized clinical trial to determine associations between patient-reported outcomes and chronologic and physiologic age in DRF management. On the basis of PC analysis, chronologic age has the strongest correlation with MHQ outcomes in the casting cohort, suggesting chronologic age is a potential proxy for functional recovery in older adults who are conservatively managing DRFs. However, measures of physiologic age, such as activity level, had a more substantial association with DRF outcomes in the surgical and VLP cohorts. This analysis demonstrates that individuals with higher activity levels have faster recovery of patient-reported outcomes after surgical intervention regardless of their chronologic age. In addition, individuals with more than 3 comorbidities have poorer outcomes after casting compared with individuals with no comorbidities. Therefore, we recommend encompassing baseline activity level and number of comorbidities into personalized treatment decisions in older adults with DRFs. For individuals who undergo casting, activity status had no significant effect on overall recovery based on MHQ scores; therefore, preinjury physiologic health status assessment is less critical in this cohort.

This study suggests that chronologic age is a poor proxy for functional demand. Increased chronologic age was associated with an increase in MHQ scores in the surgery cohort and a decrease in MHQ scores in the casting cohort. It is counterintuitive to expect increased chronologic age would result in improved MHQ scores in the surgical cohort, which further illustrates that age is a poor estimator of physiologic health. In addition, many guidelines use a chronologic age of 65 years as a proxy for low functional demand. However, evidence from this analysis demonstrates that clinically significant changes in patient-reported outcomes are observed in individuals 15 years older than baseline. In this study, a 75-year-old individual had a clinically significant difference in MHQ score compared with a 60-year-old individual; however, the difference between 60 years and 65 years was minimal. Our results indicate that further investigation is needed to develop a more efficient method for estimating a patient’s health status. Existing instruments, such as RAPA, are excellent tools to stratify by functional demand; however, RAPA does not incorporate comorbidities. Similarly, the modified frailty index (mFI) was developed to estimate functional status (independent, partial dependent, or fully dependent), but it does not include certain comorbidities, such as osteoporosis.^34^ Although the mFI has limitations, it is one of the few questionnaires that combines functional demand and comorbidities to stratify individuals by surgical risk. Therefore, we recommend hand surgeons use the mFI to assist with clinical decision-making until further studies better categorize the characteristics encompassing physiologic age.

Sustaining a DRF may impose severe restrictions on lifestyle for those who are active despite their chronologic age. These individuals can benefit from surgical treatment, which enables earlier return to daily function. Individuals with lower functional status or who live sedentary lifestyles are best treated with casting because they can still achieve recovery similar to that of patients who underwent surgery, albeit at a slower pace. Older adults are also limited by fewer remaining years of good health. The World Health Organization has noted healthy life expectancy has not increased at the same rate as overall life expectancy, placing even more value on each additional year of good health in older adults.^35^ Therefore, the earlier recovery afforded by DRF surgery may significantly improve quality of life compared with waiting a full year to achieve similar results with casting.

To our knowledge, no study has investigated the breadth of factors that could represent a patient’s physiologic age; this topic should be a focus of future investigation. This analysis is a paradigm shift in the treatment of older adults with DRFs to consider personal attributes of a patient rather than a general data point, such as age. Evidence from other surgical specialties, such as cardiac, orthopedic, and thoracic surgery, has demonstrated that increased frailty is associated with worse postoperative outcomes. However, the overall interaction between chronologic age and frailty has not been fully characterized in the hand surgery literature.^36^ Current theories suggest the improved surgical outcome for patients with increased activity level could be related to decreased skin aging and increased wound healing in the active, healthy population.^37,38,39^ These individuals are less likely to develop chronic nonhealing wounds and may be better able to tolerate and recover from surgery. In addition, increased activity is associated with improved bone health and decreased risk of osteoporosis in the aging population.^40^ Other factors for earlier recovery may be related to psychosocial factors, such as motivation and rehabilitation efforts to return to preinjury activity levels. Postoperative social support from family and friends is crucial for satisfactory recovery after many orthopedic procedures.^41^ Recovery after treatment is thus facilitated by both physical and psychosocial factors, which may contribute to the accelerated recovery in active older patients. Finally, a previous study^17^ on DRFs found that hypertension is highly associated with complication rates after surgical treatment. Further investigation with larger sample sizes is needed to identify other relevant comorbidities, which may be more highly associated with DRF outcomes after treatment.

### Limitations

This study has several limitations. First, there is no accepted physiologic age index to stratify patients by health status. Consequently, the proxy variables for physiologic age in this study were limited to those reported in the literature.^15,16,17,18^ Despite this limitation, our study identified factors that may better serve as proxies for functional demand than chronologic age and is hypothesis-generating for future discussions of appropriate guidelines for DRF treatment. A second limitation is that direct comparisons of measurements with different data types and scales (age is a continuous variable spanning from 60 to 97 years, RAPA is a categorical variable with 2 levels, and comorbidities are a continuous variable between 0 and 12) can affect the statistical and clinical significances of the results. However, the PC analysis adjusts for differences in data types and scales and mitigates for potential confounding. Third, there are inherent WRIST data set limitations. The WRIST trial was not designed to study the concept of physiologic age and may not have collected data on confounding variables, such as bone mineral density, present among the cohorts. Study participants could opt for casting instead of being randomly assigned to any of the 4 treatment groups, which could have resulted in confounding. However, the model controlled for treatment effect to minimize confounding, and WRIST was one of the largest studies conducted on DRFs in older adults, providing valuable data for interpretation despite the limitations of the data set.

## Conclusions

This secondary analysis of WRIST found that highly active older adults with DRFs should consider undergoing surgery rather than casting based on earlier postoperative recovery. Surgeons should assess a patient’s physiologic age to determine optimal DRF treatment in the elderly population. Advanced chronologic age should not be a deterrent to surgical fixation in a patient with an active lifestyle. Future studies should characterize other patient characteristics that could be associated with a patient’s physiologic age.

## Notes

Supplement 1.
Trial Protocol

Supplement 2. **eTable.** Effect of Chronologic Age, Number of Comorbidities, and Activity Level on MHQ Score at Each Time Point for Volar Lock Plating Compared to Casting

Supplement 3.
Data Sharing Statement
