# Supplementary material for: Comparison of Distal Radius Fracture Outcomes in Older Adults Stratified by Chronologic vs Physiologic Age Managed With Casting vs Surgery
Source: JAMA Netw Open. 2023 Feb 13;6(2):e2255786. doi: 10.1001/jamanetworkopen.2022.55786 (PMC9926326; doi:10.1001/jamanetworkopen.2022.55786)
Supplement: Supplement 2. — eTable. Effect of Chronologic Age, Number of Comorbidities, and Activity Level on MHQ Score at Each Time Point for Volar Lock Plating Compared to Casting [file jamanetwopen-e2255786-s002.pdf]

Supplementary Online Content

Jayaram M, Wu H, Yoon AP, Kane RL, Wang L, Chung KC. Comparison of distal radius fracture outcomes in older adults stratified by chronologic vs physiologic age managed with casting vs surgery. *JAMA Netw Open*. 2023;6(2):e2255786.
doi:10.1001/jamanetworkopen.2022.55786

eTable. Effect of Chronologic Age, Number of Comorbidities, and Activity Level on MHQ Score at Each Time Point for Volar Lock Plating Compared to Casting

This supplementary material has been provided by the authors to give readers additional information about their work.

© 2023 Jayaram M et al. *JAMA Network Open.*

eTable. Effect of Chronologic Age, Number of Comorbidities, and Activity Level on MHQ Score at Each Time Point for Volar Lock Plating Compared to Casting

	Chronologic Age			Number of Comorbidities			Activity Level		
	Effect on MHQ Score	Standard Error	P-Value	Effect on MHQ Score	Standard Error	P-Value	Effect on MHQ Score	Standard Error	P-Value
**VLP**									
**6 weeks**	0.57	0.31	.07	-0.4	1.3	.77	10.77	5.59	.05
**3 months**	0.46	0.31	.14	0.5	1.3	.68	12.46	5.64	.03*
**6 months**	0.49	0.31	.12	-0.2	1.3	.88	11.14	5.81	.06
**12 months**	0.41	0.32	.19	-0.6	1.3	.64	16.70	6.08	.006**
**Casting**									
**6 weeks**	-0.20	0.18	.27	-1.8	0.7	.01*	0.07	3.84	.99
**3 months**	-0.25	0.18	.18	-2.0	0.8	.009**	-0.72	3.88	.85
**6 months**	-0.40	0.19	.04*	-2.4	0.8	.002**	-3.64	4.14	.38
**12 months**	-0.32	0.20	.12	-2.0	0.9	.02*	-5.53	4.46	.22

*Statistical significance p<0.05
** Statistical significance p <0.01
The model is controlled for smoking, gender, race, and treatment type.

© 2023 Jayaram M et al. *JAMA Network Open.*
