# Supplementary material for: Comparison of Distal Radius Fracture Outcomes in Older Adults Stratified by Chronologic vs Physiologic Age Managed With Casting vs Surgery
Source: JAMA Netw Open. 2023 Feb 13;6(2):e2255786. doi: 10.1001/jamanetworkopen.2022.55786 (PMC9926326; doi:10.1001/jamanetworkopen.2022.55786)
Supplement: Supplement 3. — Data Sharing Statement [file jamanetwopen-e2255786-s003.pdf]

Data Sharing Statement

Jayaram. Comparison of Distal Radius Fracture Outcomes in Older Adults Stratified by Chronologic vs Physiologic Age Managed With Casting vs Surgery. *JAMA Netw Open*. Published February 13, 2023. doi:10.1001/jamanetworkopen.2022.55786

Data
Data available: No
